# Risk of QT prolongation through drug interactions between hydroxychloroquine and concomitant drugs prescribed in real world practice

**DOI:** 10.1038/s41598-021-86321-z

**Published:** 2021-03-25

**Authors:** Byung Jin Choi, Yeryung Koo, Tae Young Kim, Wou Young Chung, Yun Jung Jung, Ji Eun Park, Hong-Seok Lim, Bumhee Park, Dukyong Yoon

**Affiliations:** 1 Department of Biomedical Informatics, Ajou University School of Medicine, 206, World cup-ro, Yeongtong-gu, Suwon, Gyeonggi-do 16499 Republic of Korea; 2 Department of Pulmonology and Critical Care Medicine, Ajou University School of Medicine, Suwon, Gyeonggi-do Republic of Korea; 3 Department of Cardiology, Ajou University School of Medicine, Suwon, Gyeonggi-do Republic of Korea; 4 Office of Biostatistics, Medical Research Collaborating Center, Ajou Research Institute for Innovative Medicine, Ajou University Medical Center, Suwon, Gyeonggi-do Republic of Korea; 5 Department of Biomedical Systems Informatics, Yonsei University College of Medicine, Yongin, Gyeonggi-do Republic of Korea

**Keywords:** Adverse effects, Epidemiology

## Abstract

Hydroxychloroquine has recently received attention as a treatment for COVID-19. However, it may prolong the QTc interval. Furthermore, when hydroxychloroquine is administered concomitantly with other drugs, it can exacerbate the risk of QT prolongation. Nevertheless, the risk of QT prolongation due to drug-drug interactions (DDIs) between hydroxychloroquine and concomitant medications has not yet been identified. To evaluate the risk of QT prolongation due to DDIs between hydroxychloroquine and 118 concurrent drugs frequently used in real-world practice, we analyzed the electrocardiogram results obtained for 447,632 patients and their relevant electronic health records in a tertiary teaching hospital in Korea from 1996 to 2018. We repeated the case–control analysis for each drug. In each analysis, we performed multiple logistic regression and calculated the odds ratio (OR) for each target drug, hydroxychloroquine, and the interaction terms between those two drugs. The DDIs were observed in 12 drugs (trimebutine, tacrolimus, tramadol, rosuvastatin, cyclosporin, sulfasalazine, rofecoxib, diltiazem, piperacillin/tazobactam, isoniazid, clarithromycin, and furosemide), all with a *p* value of < 0.05 (OR 1.70–17.85). In conclusion, we found 12 drugs that showed DDIs with hydroxychloroquine in the direction of increasing QT prolongation.

## Introduction

The COVID-19 pandemic has caused more than 14 million cases of the disease worldwide as of July 22, 2020^1^, and to the best of our knowledge, no drug has been proven to target this virus to date. Meanwhile, one of the many attempts with off-label practice that received attention for treating COVID-19 was the use of hydroxychloroquine, a conventional antimalarial treatment^2,3^.

However, several studies have reported the cardiotoxic side effects of hydroxychloroquine^4^. Furthermore, when hydroxychloroquine was administered together with other drugs such as azithromycin, it can exacerbate the risk of QT prolongation due to drug-drug interactions (DDIs)^5–8^.

Hydroxychloroquine is also commonly used for some chronic diseases such as rheumatoid arthritis^9^ or systemic lupus erythematosus^10,11^. In these chronic diseases, hydroxychloroquine is usually taken over a long term, so it is often used in combination with other drugs. However, the risk of QT prolongation caused by DDIs between hydroxychloroquine and other medications has not yet been assessed comprehensively. The lack of evidence on the level of QT prolongation risk caused by DDIs poses a challenge to both physicians and regulators in treating patients.

To analyze the risk of QT prolongation caused by DDIs retrospectively from the perspective of real-world data, a large amount of electrocardiogram (ECG) results and drug prescription records are needed. Drug prescription data are usually easily accessible in electronic medical records (EMR) but extracting QTc interval information from the ECG results stored in hospital information systems could be a barrier to conducting large-scale studies with ECG data. We have made an effort to collect portable ECG results from inpatients and outpatients for the last few years^12^. In this database, ECG parameters such as RR, PR, QRS, QT, and QTc intervals were extracted from raw ECG signals. The ECG database enabled us to conduct a study to provide direct evidence of QT prolongation caused by DDIs between hydroxychloroquine and other co-medications.

In this study, we aimed to evaluate the risk of QT prolongation caused by DDIs between hydroxychloroquine and other frequently used co-medications in real-world practice by conducting large-scale retrospective case–control studies.

## Methods

This retrospective study was conducted based on EMR data. The Institutional Review Board of Ajou University Hospital approved the study (IRB No. AJIRB-MED-MDB-19-406) and waived the requirement for informed consent because only anonymized data were used retrospectively. All research was performed in accordance with relevant guidelines and regulations.

### Data sources

We used the EMR database of Ajou University Hospital, a tertiary teaching hospital in Korea, recorded between January 1996 and May 2018. The database included 177,841,556 prescriptions, 379,994,144 laboratory test results, and 3,024,891 patient demographics.

**Figure 1:**
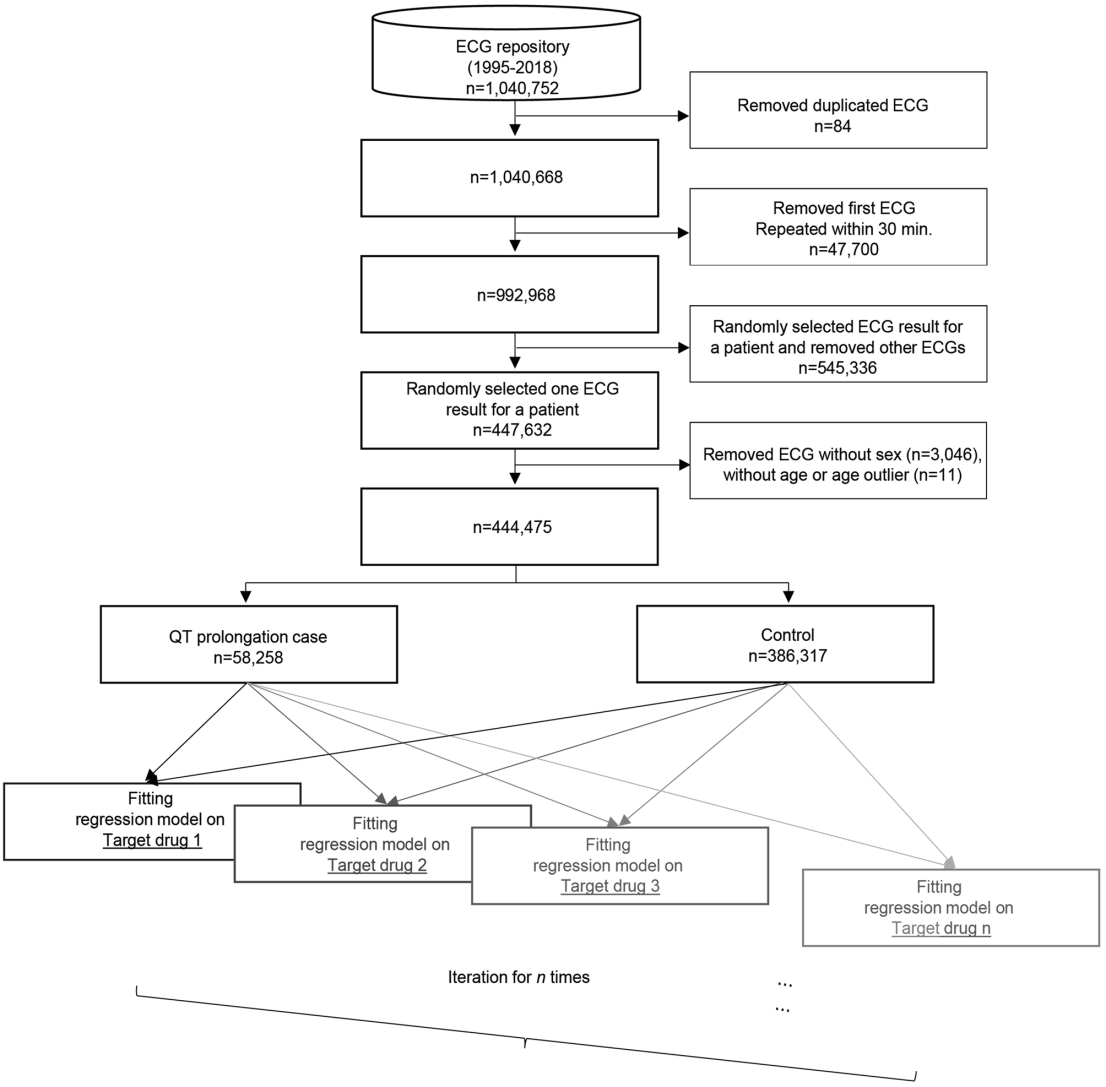
Overview of the study process. Among 1,040,752 ECG results, 992,968 ECGs that have no duplicated or repeated measurements were extracted and selected one ECG for one patient with sex and age information with no age outlier. Total of 444,475 ECG results were enrolled and divided into QT prolongation case (n = 58,258) vs. control (n = 386,317). Multiple logistic regression analysis was repeated iteratively for each target drugs.

QTc values from the same observational period were extracted from the local ECG repository in the MUSE system^12,13^. The ECG report typically contains both alphanumeric values and waveform graphs. The QTc data were extracted by parsing alphanumeric data from the PDF data extracted from the ECG repository using the web-scraping technique in our previous study^12^. This ECG database contains 1,040,752 ECG results from 447,632 patients as of January 2018 (Fig. 1). Patients whose clinical and ECG data were recorded in both EMR and our ECG repository were finally enrolled in this analysis.

### Study design and population

This study consisted of a series of retrospective case–control studies. In each study, we assessed the risk of QT prolongation due to DDI between hydroxychloroquine and one of the drugs (target drug) concomitantly used. We iterated this process for selected candidate drugs used in the subject hospital. The differences in drug use between patients with QT prolongation and those without QT prolongation were compared (Fig. 1). QT prolongation was defined as an event where corrected QT (QTc), calculated using the Bazett formula, was greater than 450 ms for males and 460 ms for females.

We excluded the following ambiguous ECG results: (1) automatically duplicated ECG measurements known as systemic errors (n = 84), and (2) sequentially measured ECG performed within 30 min, because these may reflect that the previous measurement could be an error (n = 47,700). We then randomly selected one ECG result per patient (n = 447,632). ECGs without sex or age information in the EMR and ECGs with age outliers (age < 0 and age > 120) were removed (n = 3057).

### Selection of candidate drugs for DDI risk analysis

To select candidate drugs, we first extracted all drugs prescribed concomitantly with hydroxychloroquine in the window of interest (from 7 days prior to the date of ECG measurement) from the EMR. Then, drugs used concomitantly more than 10 times were included in the analysis. The list of selected drugs and their frequency of concurrent use are provided in Supplementary Table S1 online.

### Definition of covariates

The following variables were included in the analysis to adjust for possible confounding factors on QT prolongation: (1) demographic information including sex and age at the ECG examination date, (2) comorbidities recorded in the EMR within a year before the ECG measurement date based on the International Classification Disease-10 (ICD-10). These comorbidities included myocardial infarction, congestive heart failure, ischemic stroke, hemorrhagic stroke, diabetes mellitus, hypothyroidism, renal disease, AIDS/HIV, alcohol abuse, drug abuse, liver disease, and severe liver disease; (3) the latest serum potassium and calcium levels within a year before the ECG measurement date. In patients without laboratory test results within 1 year before the ECG examination date, we imputed the missing values using the median value from patients of the same age group divided by 10-year intervals, and (4) the use of other drugs known to increase the risk of QT prolongation prescribed within 7 days before ECG measurement. The list of medications and comorbidities applied as parametric covariates is shown in Supplementary Table S2 online.

### Subgroup analysis

Because the risk of QT prolongation varies according to age and sex, we conducted a subgroup analysis. For this, we divided the subjects by sex (male and female) and age (age equal to or under 60 and age over 60). In this subgroup analysis, we included all drugs that were included in the main analysis.

### Statistical analysis

We compared the demographic characteristics of the subjects, laboratory test results, comorbidities present, medication use, and year of the ECG measurement between the QT prolongation cases and controls using Pearson's chi-square tests (for categorical data) and independent two-sample t tests (for continuous data).

We adopted multiple logistic regression with interaction terms to investigate the DDI. The detailed logit model for the *j*th drug in the candidate drug list is as follows:

$$\mathrm{Logit}\left[{P}_{j}({Y}_{i}=1)\right]=\alpha +{\beta }_{1}{x}_{i}^{(chl)}+{\beta }_{2}\left({x}_{i}^{(chl)}\times {x}_{i}^{({drug}_{j})}\right)+{\beta }_{3}{x}_{i}^{({drug}_{j})}+\sum_{k=1}^{q}{\gamma }_{k}{x}_{ik}^{(cov)}$$

where $P$_*j*_(*Y*_*i*_ = 1) is the *i*th subject's QT prolongation probability, α is the intercept, ${x}_{i}^{(chl)}$ is the *i*th study subject's use of hydroxychloroquine, ${x}_{i}^{({drug}_{j})}$ is the *i*th study subject's use of *j*th drug. ${x}_{i}^{(chl)}\times {x}_{i}^{({drug}_{j})}$ is the interaction term between ${x}_{i}^{(chl)}$ and ${x}_{i}^{({drug}_{j})}$, and ${x}_{ik}^{(cov)}$ is the *k*th covariate of the *i*th subject.

In the regression model, we calculated the coefficient of the interaction term (*β*_3_), odds ratio, and *p* value. When the *p* value of *β*_3_ was less than 0.05, we judged that hydroxychloroquine and the *i*th candidate drug had a statistically significant DDI.

For all drugs in the candidate drug lists, the multiple logistic regression was repeated iteratively to investigate which drugs showed a statistically significant DDI with hydroxychloroquine.

To visualize the interactions of statistically significant DDI drugs, we plotted the changes in the logit value of QT prolongation (Y-axis) according to the use of the target drug (X-axis) and that of hydroxychloroquine.

### Software

Data management was performed using Azure data studio version 1.19.0, and all statistical analyses were conducted using Python version 3.7 and the Python package Statsmodel version 0.11.1. The Python packages Matplotlib version 3.2.2, and seaborn Python packages version 0.10.0 were also used for visualization of the data and results.

## Results

### Baseline characteristics

The total number of subjects included in the final analysis was 444,575. Among them, the QT prolongation case group had 58,258 subjects and the control group had 386,317 subjects. Of the total patients, 218,997 were men and 225,578 were women. 1417 patients received hydroxychloroquine within 7 days before their ECG examination date. The baseline characteristics of the patient group are summarized in Table 1.

**Table 1:** Baseline characteristics of the study group.

	QT prolongation		*p* value
	Case	Control	
Total	58,258	386,317	
Hydroxychloroquine, n (%)	266 (0.5)	1151 (0.3)	< 0.001
**Sex**			< 0.001
Men, n (%)	29,932 (51.4)	189,065 (48.9)	
Women, n (%)	28,326 (48.6)	197,252 (51.1)	
Age, mean (SD)	54.9 (20.8)	42.5 (20.4)	< 0.001*
**Age, n (%)**			< 0.001
–29	102,645 (26.6)	6996 (12.0)	
30–39	68,163 (17.6)	5417 (9.3)	
40–49	69,555 (18.0)	8289 (14.2)	
50–59	59,192 (15.3)	10,355 (17.8)	
60–69	47,296 (12.2)	10,767 (18.5)	
70–79	30,296 (7.8)	11,027 (18.9)	
–80	9170 (2.4)	5407 (9.3)	
**Laboratory test result**			
Potassium, mean (SD)	4.0 (0.6)	4.1 (0.4)	< 0.001*
Calcium, mean (SD)	8.9 (0.7)	9.2 (0.5)	< 0.001*
**Comorbidity**			
Myocardial infarction, n (%)	1733 (3.0)	3519 (0.9)	< 0.001
Congestive heart failure, n (%)	1795 (3.1)	2397 (0.6)	< 0.001
Ischemic stroke, n (%)	2462 (4.2)	6366 (1.6)	< 0.001
Hemorrhagic stroke, n (%)	1506 (2.6)	2199 (0.6)	< 0.001
Diabetes mellitus, n (%)	3341 (5.7)	12417 (3.2)	< 0.001
Hypothyroidism, n (%)	246 (0.4)	1977 (0.5)	0.005
Renal disease, n (%)	2172 (3.7)	3022 (0.8)	< 0.001
AIDS/HIV, n (%)	27 (0.0)	125 (0.0)	0.114
Obesity, n (%)	94 (0.2)	2285 (0.6)	< 0.001
Drug abuse, n (%)	194 (0.3)	390 (0.1)	< 0.001
Liver disease, n (%)	1972 (3.4)	2703 (0.7)	< 0.001
**Year of QTc diagnosis (ref. year 1995–1999)**			
1995–1999	28,246 (7.3)	1921 (3.3)	
2000–2004	69,420 (18.0)	5172 (8.9)	
2005–2009	89,273 (23.1)	10,262 (17.6)	
2010–2014	102,983 (26.7)	16,396 (28.1)	
2015–2019	96,395 (25.0)	24,507 (42.1)	

Table shows baseline characteristics comparison between a group with QT prolongation and a group without QT prolongation. *Mark indicates that the p value was calculated through the two-sided T-test. Other variables were compared using chi-square tests.

### Drug–drug interaction analysis

We analyzed drugs through iterative multiple regression analysis with the interaction terms. As shown in Table 2, the ORs for the interaction terms were significant in the analysis of trimebutine, tacrolimus, tramadol, rosuvastatin, cyclosporin, sulfasalazine, rofecoxib, diltiazem, piperacillin/tazobactam, isoniazid, clarithromycin, and furosemide.

**Table 2:** Odds ratio of single drug use and interaction with hydroxychloroquine from each multiple logistic regression.

	Single drug use			Interaction with hydroxychloroquine		
	OR	95% CI	*p* value	OR	95% CI	*p* value
Trimebutine	0.84	0.76–0.94	< 0.001	2.17	1.33–3.53	< 0.001
Tacrolimus	0.75	0.58–0.97	0.03	2.27	1.31–3.93	< 0.001
Tramadol	0.98	0.90–1.07	0.66	1.70	1.24–2.34	< 0.001
Rosuvastatin	0.93	0.86–1.01	0.07	2.80	1.40–5.58	< 0.001
Cyclosporin	0.52	0.38–0.70	< 0.001	8.06	3.05–21.29	< 0.001
Sulfasalazine	0.81	0.57–1.16	0.25	2.12	1.19–3.80	0.01
Rofecoxib	0.35	0.12–0.97	0.04	8.89	1.56–50.7	0.01
Diltiazem	0.84	0.77–0.90	< 0.001	3.15	1.29–7.72	0.01
Piperacillin	1.34	1.16–1.56	< 0.001	17.85	2.10–151.94	0.01
Isoniazid	1.20	0.98–1.47	0.08	3.89	1.17–12.98	0.03
Clarithromycin	1.45	1.26–1.68	< 0.001	3.17	1.08–9.28	0.04
Furosemide	1.72	1.63–1.82	< 0.001	2.03	1.01–4.08	0.05

Detailed analysis results of 12 drugs whose interaction term with hydroxychloroquine was statistically significant (*p* > *0.05*) in linear regression. ‘Single drug use’ columns show the Odds ratio (OR), 95% Confidence interval (95% CI), and *p value* of the analyzed drug in the linear regression, and ’Interaction with hydroxychloroquine’ columns show OR, 95% CI, and the *p value* of the interaction term with hydroxychloroquine. *p value* was calculated through the two-sided T test.

In 8 of these 12 drugs (trimebutine, tramadol, rosuvastatin, cyclosporin, sulfasalazine, rofecoxib, diltiazem, and isoniazid), DDIs were present in the direction of increasing the risk of QT prolongation, even though the risk of QT prolongation was not observed with the use of these drug alone. In piperacillin, clarithromycin, and furosemide, we observed a statistically significantly higher risk of QT prolongation even when they were used alone, where DDIs were also observed. The results of the analysis of all medications are included in Supplementary Table S3 online. The full result including ORs of all covariates on tramadol is presented in Supplementary Table S4 online as an example of iterative multiple logistic regression.

**Figure 2:**
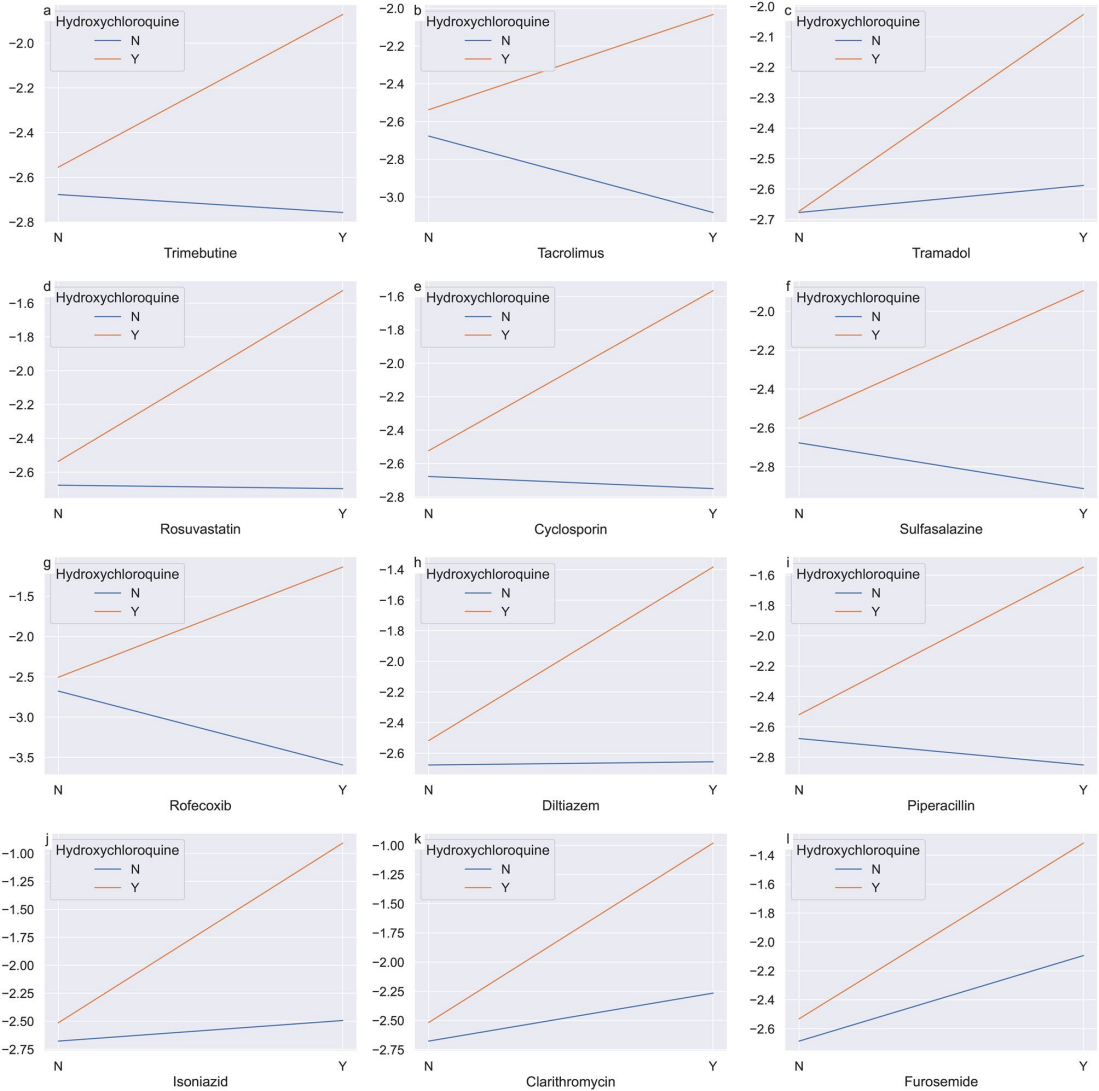
Interaction plots for 12 drugs that showed significant DDI with hydroxychloroquine. These plots show how the logit value (Y-axis) changes when the target drug (X-axis) is used alone and in combination with hydroxychloroquine. The 12 drugs plotted were drugs with statistically significant drug-drug interaction (DDI) in the iterative multiple logistic regression analysis. If there is a DDI, the two graphs have non-parallel slopes. In particular, when the interval between slopes increases, DDI exists in the direction of increasing QT prolongation. The interval between slopes increases in all 12 drugs.

In each regression model, the average logit value of the intercept was − 2.7. This corresponds to a QT prolongation probability of 0.07 when all other variables were set as 0 (i.e., having no risk factor and no exposure to both hydroxychloroquine and target drug). The average logit value when using hydroxychloroquine alone without any other risk factors was − 2.5 (QT prolongation probability of 0.08). As shown in Fig. 2, the logit value (probability of QT prolongation occurrence) increased when hydroxychloroquine and the target drug were administered at the same time compared to expected additive effect of the two drugs in all 12 drugs. This means that all 12 drugs have DDIs in the direction of increasing the risk of QT prolongation.

### Subgroup analysis

We divided the patient group into four subgroups according to sex and age. We then investigated whether the drugs used in each subgroup showed significant (*p* < 0.05) DDIs with hydroxychloroquine. We found that five drugs (tramadol, trimebutine, nifedipine, sulfasalazine, and rofecoxib) showed significant (*p* < 0.05) DDIs with hydroxychloroquine in the female subgroup, and three drugs (tacrolimus, paracetamol, and propacetamol) showed significant (*p* < 0.05) DDIs in the male patient group. Nine drugs (rosuvastatin, tacrolimus, sulfasalazine, trimebutine, ranitidine, ranitidine, diltiazem, celecoxib, paracetamol, and aceclofenac) showed DDIs with hydroxychloroquine in the group under 60 years of age. In the elderly group over 60 years old, no drug was significant (*p* < 0.05), but five drugs (clopidogrel, bisoprolol, furosemide, tramadol, and trimebutine) implied the possibility of a DDI (*p* < 0.1). The ten drugs with the highest probability of interaction for each subgroup are described in Table 3.

**Table 3:** Ten drugs with the lowest *p* value of interaction terms for each subgroup.

	Single drug use			Interaction with hydroxychloroquine		
	OR	95% CI	*p* value	OR	95% CI	*p* value
**By age**						
Over 60						
Clopidogrel	1.013	0.947–1.083	0.708	0.42	0.175–1.009	0.052
Bisoprolol	0.856	0.734–0.997	0.046	0.127	0.015–1.055	0.056
Furosemide	1.445	1.346–1.551	< 0.0001	2.326	0.972–5.567	0.058
Tramadol	1.004	0.81–1.245	0.972	2.604	0.876–7.738	0.085
Trimebutine	0.924	0.799–1.069	0.288	1.754	0.903–3.406	0.097
Cimetidine	1.054	0.949–1.171	0.322	0.287	0.064–1.283	0.102
Amlodipine	0.94	0.867–1.02	0.136	0.551	0.247–1.231	0.146
Chlorphenamine	1.085	0.981–1.199	0.112	0.398	0.115–1.38	0.147
Diltiazem	0.813	0.739–0.894	< 0.0001	2.169	0.73–6.45	0.164
Meloxicam	0.805	0.589–1.1	0.174	1.521	0.841–2.749	0.165
Equal to or under 60						
Rosuvastatin	0.864	0.755–0.989	0.034	7.253	2.437–21.587	< 0.0001
Tramadol	0.916	0.8–1.049	0.203	2.506	1.587–3.959	< 0.0001
Sulfasalazine	0.767	0.491–1.2	0.245	3.451	1.632–7.296	0.001
Tacrolimus	0.593	0.438–0.804	0.001	3.52	1.709–7.25	0.001
Trimebutine	0.774	0.654–0.916	0.003	2.558	1.238–5.285	0.011
Ranitidine	1.199	1.103–1.303	< 0.0001	0.346	0.152–0.789	0.012
Diltiazem	0.974	0.843–1.126	0.721	5.506	1.295–23.404	0.021
Celecoxib	0.75	0.525–1.072	0.115	2.217	1.126–4.363	0.021
Paracetamol	0.961	0.879–1.05	0.375	2.148	1.095–4.215	0.026
Aceclofenac	0.739	0.572–0.954	0.02	2.146	1.071–4.299	0.031
**By sex**						
Female						
Tramadol	0.926	0.819–1.046	0.217	1.809	1.254–2.612	0.002
Trimebutine	0.837	0.72–0.973	0.02	2.29	1.344–3.905	0.002
Nifedipine	1.297	1.116–1.509	0.001	2.828	1.252–6.387	0.012
Sulfasalazine	0.801	0.477–1.345	0.402	2.508	1.179–5.334	0.017
Celecoxib	0.76	0.64–0.904	0.002	1.716	1.08–2.728	0.022
Tacrolimus	0.722	0.479–1.088	0.12	1.899	0.937–3.849	0.075
Prednisolone	0.696	0.596–0.813	< 0.0001	1.512	0.911–2.51	0.11
Furosemide	1.462	1.341–1.595	< 0.0001	1.971	0.843–4.611	0.117
Aceclofenac	0.687	0.538–0.876	0.002	1.701	0.868–3.336	0.122
Meloxicam	0.763	0.569–1.022	0.07	1.357	0.847–2.174	0.204
Male						
Tacrolimus	0.769	0.551–1.073	0.122	4.64	1.589–13.546	0.005
Paracetamol	1.145	1.048–1.252	0.003	4.139	1.375–12.456	0.012
Propacetamol	1.239	1.117–1.375	< 0.0001	7.962	1.585–40.01	0.012
Nifedipine	1.361	1.185–1.564	< 0.0001	0.208	0.044–0.974	0.046
Furosemide	1.934	1.79–2.09	< 0.0001	3.99	0.986–16.153	0.052
Sulglicotide	0.814	0.574–1.155	0.248	3.665	0.982–13.679	0.053
Atorvastatin	0.794	0.719–0.877	< 0.0001	2.919	0.953–8.942	0.061
Clopidogrel	0.992	0.923–1.067	0.831	0.368	0.09–1.505	0.164
Azathioprine	0.702	0.375–1.315	0.269	0.197	0.019–1.999	0.169
Tramadol	1.023	0.909–1.152	0.704	1.569	0.817–3.015	0.176

This table shows the ten drugs with the lowest *p* value of the interaction term with hydroxychloroquine in each subgroup. Each subgroup was divided into over 60 years old and under 60 years old or divided into male and female. ‘Single drug use’ columns show the odds ratio (OR), 95% confidence interval (95% CI), and *p* value of the analyzed drug in the linear regression, and ‘Interaction with hydroxychloroquine’ columns show OR, 95% CI, and the *p* value of the interaction term with hydroxychloroquine. *p* value was calculated through the two-sided T test.

## Discussion

Using EMRs obtained at a tertiary hospital, we investigated DDIs between hydroxychloroquine and 118 other drugs. We observed significant (*p* < 0.05) DDIs in 12 drugs. Among them, piperacillin/tazobactam, clarithromycin, and furosemide showed a risk of QT prolongation in individual treatment, and a DDI in the direction of increasing QT prolongation risk was also observed. However, for eight drugs (trimebutine, tramadol, rosuvastatin, cyclosporin, sulfasalazine, rofecoxib, diltiazem, and isoniazid), DDI was present in the direction of increasing the risk of QT prolongation, even though the QT prolongation risk of individual drugs was not significant (*p* < 0.05).

It is well known that hydroxychloroquine can cause QT prolongation^14,15^. With the concern that DDIs between hydroxychloroquine and other drugs may exacerbate side effects such as this, several studies on the DDI of hydroxychloroquine have been investigated^14,16,17^. However, existing studies have selectively studied the DDI between hydroxychloroquine and some drugs that captured the attention of clinicians, such as immunosuppressants or antibiotics^16–19^.

In the case of patients with severe COVID-19, dozens of drugs are prescribed simultaneously with hydroxychloroquine, not only immunosuppressants or antibiotics. These drugs are medications that are taken regularly to treat chronic underlying diseases or are prescribed to relieve the patient's symptoms without specific indications. Most of these drugs are known to have minimal effects on QT prolongation when administered individually, but there is a risk of prolonging the QT interval indirectly through DDI with hydroxychloroquine. To overcome this problem, we conducted a DDI study not only on selected drugs but for all drugs that were prescribed concurrently with hydroxychloroquine in the chosen period. Consequently, the risk of prolonging the QT interval through DDI was observed in 12 drugs, even though the risk of QT prolongation in individual prescriptions was observed in only three drugs.

According to the therapeutic class of these drugs, three antibiotics (clarithromycin, piperacillin, and isoniazid) showed DDIs, and the mechanisms of these three antibiotics were also varied. Clarithromycin is a macrolide, such as azithromycin. Therefore, a DDI between clarithromycin and hydroxychloroquine suggests the possibility of a DDI in the combined therapy of azithromycin and hydroxychloroquine, which was suggested as a COVID-19 treatment^20^. In this study, azithromycin was not included since there was not a sufficient number of patients who used the drug concurrently with hydroxychloroquine. DDI studies for azithromycin would be required later using a more massive clinical database so this can be investigated further.

Besides, other antibiotics can also be lethal in diseases such as COVID-19, which is deeply associated with respiratory disease. Piperacillin is a drug commonly prescribed in the hospitalization of patients in intensive care units (ICU) due to respiratory diseases such as pneumonia^21^. Isoniazid is a drug that is used to treat tuberculosis, a disease that has a harmful effect on the lungs in the long term. As with COVID-19, tuberculosis is more common in developing countries^22^. Therefore, if patients in developing countries have to treat tuberculosis and COVID-19 simultaneously, delicate QT interval monitoring is required.

Rofecoxib showed DDI with hydroxychloroquine in our main analysis, and several NSAIDs, such as celecoxib and paracetamol, showed interactions with hydroxychloroquine in the main analysis and in the young female subgroup analysis. Meanwhile, trimebutine, a spasmolytic drug commonly used for indigestion, showed DDI in the main analysis and in the young female subgroup analysis. These drugs are prescribed extensively and are often administered even in non-essential situations. Formerly, the side effects of these drugs are known to be minor. As a result, doctors are not cautious about these drugs, and they are often used as over-the-counter medicines. However, this study showed the possibility of DDI between these drugs and hydroxychloroquine, which could cause QT prolongation. Individually non-dangerous drugs could cause drug side effects due to DDI in environments where large amounts of drugs are co-administered, such as COVID-19. Unnecessary routine prescriptions should be reduced, and appropriate alternative drugs should be selected.

Tramadol, one of the most commonly prescribed opioids, also showed DDI interaction with hydroxychloroquine. The risk of QT prolongation in opioids has already been reported^23^. In particular, after COVID-19, it became accessible to purchase drugs on a non-face-to-face basis, and so opioid use disorder is rapidly increasing^24,25^. If patients with opioid use disorder are infected with COVID-19, it would be essential to prevent fatal cardiotoxic adverse effects through precise QT monitoring.

In the subgroup analysis, the drugs that showed DDIs differed substantially by subgroup. Only four drugs showed significant (*p* < 0.05) interactions in two or more subgroups among the drugs analyzed. These results suggest that the interaction of hydroxychloroquine with other drugs may vary by age and sex. However, the difference in the drugs showing interactions among subgroups could be due to the difference in prescription patterns for each subgroup. In the future, studies that focus on specific subgroups would require additional statistical methods to compensate for these prescription patterns.

It is common to manage DDIs in clinical practice, but the mechanisms of DDIs are not adequately understood. One possible hypothesis for the interaction of drugs is through the activity of the CYP 450 enzyme^26^. In the risk of QT prolongation interaction observed in this study, 7 out of 12 drugs had inhibitory effects on the metabolic pathway of hydroxychloroquine. CYP 450 2C8, and 3A4/5, especially clarithromycin and diltiazem, are known potent inhibitors of CYP 450 3A4^27^. In addition, tramadol is also known to share the CYP 450 3A4 enzyme with hydroxychloroquine^28^. These drugs may interfere with the metabolism of hydroxychloroquine, leading to QT prolongation by raising the hydroxychloroquine concentration to more than necessary.

This study has some limitations. First, it used a single institutional database. In the future, a multi-center study would be required to further generalize the results of this DDI study. Second, the drug dose information was not included in this study. If the drug dose information is used in future studies, it will be possible to investigate DDI changes according to the drug dose.

## Conclusion

The current study investigated DDIs with hydroxychloroquine in 118 drugs using real-world data. We found statistically significant DDI in 12 drugs (trimebutine, tacrolimus, tramadol, rosuvastatin, cyclosporin, sulfasalazine, rofecoxib, diltiazem, piperacillin/tazobactam, isoniazid, clarithromycin, and furosemide) and the direction of the DDI was toward increasing the risk of QT prolongation. In eight drugs (trimebutine, tramadol, rosuvastatin, cyclosporin, sulfasalazine, rofecoxib, diltiazem, and isoniazid), DDI was present in the direction of increasing the risk of QT prolongation, even though the risk of QT prolongation was not observed with the use of those drugs alone.

## Supplementary Information

Supplementary Information

## Data Availability

The datasets generated during and/or analyzed during the current study are available from the corresponding author on reasonable request.
