# Supplementary material for: Risk of QT prolongation through drug interactions between hydroxychloroquine and concomitant drugs prescribed in real world practice
Source: Sci Rep. 2021 Mar 25;11:6918. doi: 10.1038/s41598-021-86321-z (PMC7994840; doi:10.1038/s41598-021-86321-z)
Supplement: Supplementary file 1 — Supplementary Information [file 41598_2021_86321_MOESM1_ESM.pdf]

Supplementary information for

Risk of QT Prolongation through Drug Interactions between Hydroxychloroquine and Concomitant Drugs Prescribed in Real World Practice

Byung Jin Choi, Yeryung Koo, Tae Young Kim, Wou Young Chung, Yun Jung Jung,

Ji Eun Park, Hong-Seok Lim, Bumhee Park, Dukyong Yoon

Supplelementary Table S1. The list of selected drugs and their frequency of concurrent use.

Drug name	Concurrent use with HCQ	Drug name	Concurrent use with HCQ
methylprednisolone	614	clotiazepam	21
tramadol	328	Angiotensin II antagonists and Calcium channel blockers	20
meloxicam	323	itopride	20
bucillamine	259	levocetirizine	20
bucillamine	215	rabeprazole	20
celecoxib	164	dexamethasone	20
clonazepam	153	fentanyl	20
ranitidine	135	roxatidine	19
acetylsalicylic acid	121	metoclopramide	19
sulfasalazine	118	telmisartan	18
aceclofenac	105	propranolol	18
trimebutine	95	pethidine	18
tacrolimus	94	domperidone	17
Other antiinflammatory and Antirheumatic agents, non-steroids	89	esomeprazole	17
paracetamol	88	minocycline	17
deflazacort	80	clarithromycin	16
atorvastatin	78	bisoprolol	16
diacerein	69	glimepiride	16
omeprazole	67	triamcinolone	16
amlodipine	64	montelukast	16
methotrexate	64	hydrochlorothiazide	15
alprazolam	63	cefpodoxime	15
cimetidine	61	famotidine	15
levothyroxine sodium	57	esomeprazole	14
azathioprine	53	hydroxyzine	14
methotrexate	52	nimesulide	14
leflunomide	49	benzydamine	14
lansoprazole	48	ramipril	14
colchicine	46	loratadine	14
nifedipine	46	azelastine	14
chlorphenamine	43	raloxifene	14
furosemide	41	morphine	14
pantoprazole	39	duloxetine	13
amoxapine	39	pseudoephedrine, combinations	13
rosuvastatin	38	ipratropium bromide	13
clopidogrel	38	piroxicam	13
midazolam	34	isoniazid	12
ketorolac	34	mycophenolic acid	12
amitriptyline	33	ketoprofen	12
nabumetone	33	oxycodone	12
zolpidem	32	megestrol	12
cilazapril	32	lercanidipine	12
fexofenadine	32	theophylline	12
tianeptine	31	metoprolol	12
alendronic acid and colecalciferol	30	beraprost	12
valsartan	28	norepinephrine	11
misoprostol	28	codeine	11
candesartan	27	propofol	11
propacetamol	26	pitavastatin	11
ebastine	26	escitalopram	11

metformin	25	Rofecoxib	10
isosorbide dinitrate	25	piperacillin and beta-lactamase Inhibitor	10
pregabalin	25	Cyproheptadine	10
diltiazem	24	rocuronium bromide	10
carvedilol	24	tamsulosin	10
losartan	24	perindopril	10
gabapentin	23	diazepam	10
ciclosporin	21	hydrocortisone	10
trazodone	21	paroxetine	10

Supplementary Table S2. The list of medications and comorbidities applied as covariates

Category	Variables
Demographic	SEX
	AGE
	ECG measurement year
Labaratory test result	potassium
	calcium
Medications	Antiarrhythmic drugs
	Antianginal drugs
	Anticholinergic
	Antifungals
	Fluoroquinolone antibiotics
	Antihistamines
	Antineoplastic drugs
	Selective serotonin reuptake inhibitors
	Opioids
	Bronchodilators (beta-agonists)
	Antidiarrheals
	Antiemetics
	Gastrointestinal promotility
	Neurologic drugs
	Antipsychotics
	Tricyclic and tetracyclic antidepressants
	Selective serotonin reuptake inhibitors
	Vasodilator drugs
Comorbidities	Myocardial infarction
	Congestive heart failure
	Ischemic stroke
	Hemorrhagic stroke
	Diabetes mellitus
	Hypothyroidism
	Renal disease
	AIDS/HIV
	Obesity
	Drug abuse
	Liver disease

Supplementary Table S3. The analysis results of all medications.

		single drug			interaction		
Drug name	ATCCODE	Odds	CI (95%)	*p*-value	Odds	CI (95%)	*p*-value
trimebutine	A03AA05	0.84	0.76-0.94	<0.0001	2.17	1.33-3.53	<0.0001
tacrolimus	L04AD02	0.75	0.58-0.97	0.03	2.27	1.31-3.93	<0.0001
tramadol	N02AX52	0.98	0.90-1.07	0.66	1.7	1.24-2.34	<0.0001
rosuvastatin	C10AA07	0.93	0.86-1.01	0.07	2.8	1.40-5.58	<0.0001
ciclosporin	L04AD01	0.52	0.38-0.70	<0.0001	8.06	3.05-21.29	<0.0001
sulfasalazine	A07EC01	0.81	0.57-1.16	0.25	2.12	1.19-3.80	0.01
rofecoxib	M01AH02	0.35	0.12-0.97	0.04	8.89	1.56-50.7	0.01
diltiazem	C08DB01	0.84	0.77-0.90	<0.0001	3.15	1.29-7.72	0.01
piperacillin and beta-lactamase inhibitor	J01CR05	1.34	1.16-1.56	<0.0001	17.85	2.10-151.94	0.01
isoniazid	J04AC01	1.2	0.98-1.47	0.08	3.89	1.17-12.98	0.03
clarithromycin	J01FA09	1.45	1.26-1.68	<0.0001	3.17	1.08-9.28	0.04
furosemide	C03CA01	1.72	1.63-1.82	<0.0001	2.03	1.01-4.08	0.05
mycophenolic acid	L04AA06	0.3	0.21-0.43	<0.0001	4.05	1.00-16.43	0.05
esomeprazole	M01AE52	0.61	0.36-1.03	0.07	3.51	1.00-12.29	0.05
cyproheptadine	A15	1.04	0.85-1.26	0.7	3.7	0.97-14.04	0.05
bisoprolol	C07AB07	0.89	0.79-1.01	0.08	0.21	0.04-0.99	0.05
paracetamol	N02BE01	1.04	0.97-1.11	0.25	1.61	0.96-2.70	0.07
duloxetine	N06AX21	1.06	0.80-1.4	0.67	2.83	0.87-9.27	0.08
celecoxib	M01AH01	0.83	0.72-0.96	0.01	1.43	0.93-2.19	0.1
norepinephrine	C01CA03	1.19	1.08-1.31	<0.0001	3.02	0.82-11.09	0.1
zolpidem	N05CF02	1.18	1.03-1.36	0.02	0.37	0.11-1.24	0.11
codeine	R05DA04	1.5	1.33-1.70	<0.0001	0.16	0.02-1.58	0.12
pseudoephedrine, Combinations	R01BA52	1.25	1.05-1.49	0.01	2.58	0.79-8.46	0.12
amlodipine	C08CA01	0.99	0.92-1.06	0.7	0.57	0.27-1.17	0.13
clopidogrel	B01AC04	1.03	0.97-1.09	0.37	0.53	0.23-1.21	0.13
rocuronium bromide	M03AC09	0.86	0.76-0.98	0.02	2.75	0.73-10.35	0.13
leflunomide	L04AA13	0.68	0.34-1.37	0.28	2.08	0.78-5.51	0.14
omeprazole	A02BC01	0.84	0.72-0.98	0.03	1.56	0.84-2.91	0.16
candesartan	C09CA06	1.08	0.97-1.21	0.16	1.85	0.78-4.42	0.17
angiotensin II antagonists and calcium channel blockers	C09DB	0.83	0.67-1.02	0.08	1.98	0.72-5.45	0.18
cilazapril	C09AA08	1.22	1.00-1.48	0.05	0.43	0.12-1.49	0.18
hydroxyzine	N05BB01	0.77	0.38-1.54	0.46	0.25	0.03-1.99	0.19
prednisolone	H02AB06	0.76	0.69-0.85	<0.0001	1.31	0.87-1.99	0.2
methotrexate	L01BA01	1.21	0.64-2.26	0.56	0.47	0.15-1.48	0.2
propacetamol	N02BE05	1.2	1.11-1.3	<0.0001	1.72	0.75-3.94	0.2
aceclofenac	M01AB16	0.68	0.57-0.82	<0.0001	1.46	0.82-2.61	0.2
fexofenadine	R06AX26	1.04	0.90-1.2	0.62	0.46	0.14-1.56	0.21
nimesulide	M01AX17	0.82	0.68-0.98	0.03	2.37	0.60-9.37	0.22
midazolam	N05CD08	1.18	1.09-1.27	<0.0001	1.6	0.74-3.46	0.23
amitriptyline	N06AA09	1.35	1.07-1.69	0.01	0.51	0.16-1.59	0.24
ketoprofen	M02AA10	1.19	0.75-1.89	0.46	0.3	0.04-2.52	0.27
metformin	A10BA02	0.99	0.90-1.09	0.9	1.66	0.68-4.08	0.27
chlorphenamine	R06AB04	1.21	1.12-1.30	<0.0001	0.63	0.28-1.44	0.28
meloxicam	M01AC06	0.76	0.59-0.97	0.03	1.25	0.83-1.90	0.29
carvedilol	C07AG02	1.15	1.06-1.24	<0.0001	1.63	0.65-4.08	0.3
ipratropium bromide	R03BB01	1.57	1.39-1.77	<0.0001	1.83	0.55-6.07	0.32
glimepiride	A10BB12	1.03	0.93-1.13	0.6	0.46	0.10-2.12	0.32
atorvastatin	C10AA05	0.87	0.81-0.93	<0.0001	1.32	0.76-2.27	0.32
ranitidine	A02BA02	1.15	1.09-1.22	<0.0001	0.79	0.50-1.27	0.33
roxatidine	A02BA06	1.43	1.13-1.82	<0.0001	0.37	0.05-2.90	0.34

itopride	A03FA07	1.01	0.89-1.14	0.9	1.64	0.59-4.60	0.34
benzydamine	A01AD02	1.14	0.99-1.31	0.08	0.47	0.10-2.21	0.34
acetylsalicylic acid	B01AC06	1.01	0.96-1.06	0.69	1.25	0.79-1.98	0.34
alendronic acid and colecalciferol	M05BB03	0.67	0.46-0.98	0.04	1.57	0.61-4.00	0.35
propofol	N01AX10	0.18	0.15-0.22	<0.0001	1.94	0.49-7.71	0.35
triamcinolone	H02AB08	0.63	0.50-0.80	<0.0001	1.84	0.50-6.82	0.36
oxycodone	N02AA05	1.04	0.91-1.19	0.57	1.8	0.51-6.39	0.36
hydrochlorothiazide	C03AA03	1.15	1.03-1.28	0.01	1.64	0.54-5.04	0.38
methotrexate	L04AX03	0.66	0.29-1.47	0.3	1.58	0.56-4.42	0.38
ramipril	C09AA05	1.43	1.29-1.59	<0.0001	0.54	0.13-2.31	0.4
levocetirizine	R06AE09	0.92	0.77-1.11	0.39	0.56	0.12-2.50	0.44
telmisartan	C09CA07	0.89	0.79-1.01	0.07	1.52	0.52-4.45	0.45
loratadine	R06AX13	0.86	0.65-1.14	0.28	1.71	0.43-6.75	0.45
megestrol	L02AB01	0.89	0.80-0.99	0.04	0.52	0.09-2.83	0.45
rabeprazole	A02BC04	0.73	0.60-0.90	<0.0001	1.5	0.51-4.43	0.46
colchicine	M04AC01	0.87	0.65-1.17	0.35	0.69	0.25-1.88	0.47
tianeptine	N06AX14	0.73	0.61-0.89	<0.0001	1.41	0.55-3.59	0.47
nifedipine	C08CA05	1.32	1.20-1.47	<0.0001	1.27	0.65-2.48	0.48
tamsulosin	G04CA02	0.88	0.78-0.99	0.03	0.58	0.12-2.89	0.51
cefpodoxime	J01DD13	1.17	1.04-1.32	0.01	0.63	0.17-2.43	0.51
isosorbide dinitrate	C01DA08	1.44	1.35-1.53	<0.0001	0.72	0.28-1.87	0.51
domperidone	A03FA03	0.94	0.81-1.1	0.46	0.61	0.13-2.80	0.52
deflazacort	H02AB13	0.49	0.30-0.80	<0.0001	1.34	0.54-3.29	0.53
lercanidipine	C08CA13	1.3	1.10-1.54	<0.0001	1.48	0.43-5.09	0.54
cimetidine	A02BA01	1.07	0.99-1.15	0.09	0.79	0.36-1.72	0.55
nabumetone	M01AX01	0.95	0.46-1.96	0.89	0.65	0.15-2.76	0.56
esomeprazole	A02BC05	0.92	0.75-1.13	0.43	1.36	0.45-4.13	0.58
azelastine	R06AX19	0.87	0.62-1.21	0.39	0.56	0.07-4.57	0.59
trazodone	N06AX05	1.05	0.89-1.23	0.58	1.31	0.48-3.59	0.6
valsartan	C09CA03	0.89	0.76-1.03	0.13	0.76	0.27-2.15	0.6
pantoprazole	A02BC02	1.17	1.07-1.28	<0.0001	0.82	0.38-1.77	0.61
amoxapine	N06AA17	2.2	0.78-6.24	0.14	0.72	0.2-2.59	0.61
clotiazepam	N05BA21	0.79	0.65-0.96	0.02	0.65	0.13-3.35	0.61
montelukast	R03DC03	0.93	0.78-1.11	0.42	0.69	0.15-3.15	0.63
misoprostol	A02BB01	0.99	0.81-1.23	0.96	0.73	0.19-2.77	0.64
levothyroxine sodium	H03AA01	1.01	0.90-1.13	0.89	0.84	0.40-1.76	0.64
theophylline	R03DA04	1.34	1.18-1.51	<0.0001	1.31	0.37-4.64	0.67
ketorolac	M01AB15	0.9	0.84-0.97	<0.0001	1.2	0.52-2.77	0.67
metoclopramide	A03FA01	1.13	0.98-1.30	0.1	0.8	0.26-2.43	0.69
perindopril	C09AA04	1.31	1.16-1.47	<0.0001	1.32	0.34-5.19	0.69
losartan	C09CA01	0.98	0.88-1.10	0.74	0.8	0.26-2.48	0.7
methylprednisolone	H02AB04	1.14	1.01-1.28	0.03	0.94	0.70-1.28	0.71
dexamethasone	H02AB02	1.11	0.99-1.23	0.07	0.8	0.24-2.61	0.71
pitavastatin	C10AA08	0.73	0.59-0.89	<0.0001	0.75	0.14-4.02	0.74
metoprolol	C07AB02	1.01	0.91-1.12	0.8	1.24	0.32-4.77	0.76
raloxifene	G03XC01	0.58	0.38-0.88	0.01	0.79	0.16-3.93	0.77
azathioprine	L04AX01	0.86	0.57-1.29	0.46	1.12	0.48-2.62	0.8
clonazepam	N03AE01	1.05	0.93-1.17	0.44	0.94	0.60-1.48	0.8
pregabalin	N03AX16	0.92	0.79-1.08	0.31	0.9	0.32-2.53	0.83
gabapentin	N03AX12	0.73	0.66-0.80	<0.0001	1.12	0.39-3.2	0.83
beraprost	B01AC19	1.05	0.83-1.34	0.66	0.84	0.18-4.04	0.83
fentanyl	N02AB03	1.1	1.02-1.18	0.01	1.12	0.41-3.03	0.83
propranolol	C07AA05	1.14	1.00-1.3	0.05	0.88	0.27-2.87	0.83
diacerein	M01AX21	0.93	0.69-1.25	0.63	0.93	0.47-1.85	0.84
lansoprazole	A02BC03	0.82	0.71-0.94	<0.0001	0.93	0.43-1.99	0.85
diazepam	N05BA01	0.98	0.83-1.16	0.79	1.15	0.21-6.26	0.87
hydrocortisone	H02AB09	1.4	1.20-1.64	<0.0001	1.12	0.25-5.12	0.88
famotidine	A02BA03	1.16	1.05-1.27	<0.0001	1.08	0.31-3.72	0.91

alprazolam	N05BA12	1.01	0.94-1.09	0.74	1.03	0.53-2.01	0.92
other antiinflammatory and antirheumatic agents, non-steroids	M01AX	0.77	0.62-0.96	0.02	0.97	0.54-1.77	0.93
bucillamine	M01CC02	0.98	0.59-1.63	0.94	1.02	0.54-1.90	0.96
morphine	N02AA01	1.65	1.50-1.81	<0.0001	1.01	0.32-3.16	0.98
pethidine	N02AB02	0.66	0.60-0.73	<0.0001	1.01	0.28-3.72	0.98
minocycline	J01AA08	0.61	0.34-1.08	0.09	0.99	0.12-8.23	0.99
piroxicam	M02AA07	1	0.68-1.48	0.99	0.99	0.20-4.95	0.99
ebastine	R06AX22	0.8	0.63-1.01	0.06	1	0.33-3.09	0.99
escitalopram	N06AB10	1.38	1.08-1.76	0.01	0	0.0-inf	1
paroxetine	N06AB05	0.66	0.49-0.90	0.01	0	0.0-inf	1

Supplementary Table S4. The result of analysis of tramadol

	OR	95% CI	p-value
Hydroxychloroquine	1.05	(0.88-1.24)	0.607
Tramadol	0.98	(0.9-1.07)	0.655
Interaction of Hydroxychloroquine & tramadol	1.70	(1.24-2.34)	0.001
Female (ref. male)	0.89	(0.87-0.9)	<0.001
Age (Mean/SD)	1.02	(1.02-1.02)	<0.001
Laboratory test result			
Potassium (mEq/l)	0.60	(0.59-0.61)	<0.001
Calcium (mg/dl)	0.57	(0.56-0.58)	<0.001
Drugs that known risk for QTc prolongation			
Antiarrhythmic drugs	2.57	(2.27-2.9)	<0.001
Antianginal drugs	1.69	(0.57-4.97)	0.34
Anticholinergic	1.17	(0.94-1.47)	0.159
Antifungals	1.34	(1.05-1.71)	0.017
Fluoroquinolone antibiotics	1.04	(0.95-1.13)	0.382
Antihistamines	1.41	(1.15-1.73)	0.001
Antineoplastic drugs	1.31	(1.07-1.6)	0.009
Selective serotonin reuptake inhibitors	0.98	(0.89-1.07)	0.647
Opioids	1.05	(0.73-1.53)	0.783
Bronchodilators (beta-agonists)	1.79	(1.52-2.11)	<0.001
Antidiarrheals	1.05	(0.67-1.67)	0.821
Antiemetics	0.94	(0.77-1.15)	0.537
Gastrointestinal promotility	1.23	(1.16-1.3)	<0.001
Neurologic drugs	1.09	(0.91-1.3)	0.37
Antipsychotics	1.21	(1.12-1.31)	<0.001
Tricyclic and tetracyclic antidepressants	0.94	(0.84-1.06)	0.305
Selective serotonin reuptake inhibitors	1.14	(1.01-1.29)	0.032
Vasodilator drugs	1.31	(1.17-1.46)	<0.001
Comorbidity			
Myocardial infarction	1.91	(1.79-2.03)	<0.001
Congestive heart failure	2.56	(2.39-2.74)	<0.001
Ischemic stroke	1.51	(1.43-1.59)	<0.001
Hemorrhagic stroke	3.39	(3.16-3.64)	<0.001
Diabetes mellitus	1.11	(1.06-1.16)	<0.001
Hypothyroidism	0.78	(0.67-0.9)	0.001
Renal disease	3.37	(3.16-3.59)	<0.001
AIDS/HIV	1.21	(0.78-1.86)	0.397
Obesity	0.54	(0.44-0.67)	<0.001
Drug abuse	4.00	(3.32-4.82)	<0.001
Liver disease	3.60	(3.37-3.83)	<0.001
Year of QTc diagnosis(ref.year 1995-1999)			
2000-2004	0.92	(0.87-0.97)	0.002
2005-2009	1.42	(1.35-1.49)	<0.001
2010-2014	1.95	(1.85-2.05)	<0.001
2015-2019	2.92	(2.78-3.07)	<0.001
